# Long-Term Results Following Antibiotic Treatment of Acute Appendicitis in Adults

**DOI:** 10.1007/s00268-017-3987-6

**Published:** 2017-03-24

**Authors:** K. Lundholm, J. Hansson-Assarsson, C. Engström, B.-M. Iresjö

**Affiliations:** 1 Surgical Metabolic Research Lab, Department of Surgery, Sahlgrenska University Hospital and The Institute of Clinical Sciences, University of Gothenburg, Vita Stråket 12, plan 2, 413 45 Göteborg, Sweden; 2 Department of Surgery, Kalmar County Hospital, Kalmar, Sweden

## Abstract

**Background:**

Antibiotic treatment of acute appendicitis has gained interest and inquiries. Reports have demonstrated both safety and high resolution of symptoms and inflammation following antibiotic treatment of appendicitis, but information on long-term results is required. Our present aim was therefore to evaluate long-term recurrence rate of initial antibiotics-alone treatment for suspected acute appendicitis.

**Methods:**

Patients with favourable response to antibiotics in earlier randomized (RCT, *n* = 97) and population-based (PBT, *n* = 342) studies as well as subsequently treated non-randomized (Non-R, *n* = 271) patients are evaluated for long-term risk to relapse demanding surgical appendectomy; altogether 710 patients.

**Results:**

Clinical characteristics among randomized and non-randomized patients were similar without any statistical difference according to abdominal symptoms and degree of systemic inflammation (CRP, WCC) when antibiotic treatment started. Females and males showed the same results. The median follow-up time was 2162 days (5.92 years), and the range across highest and lowest follow-up was 3495 days (range 2–3497) for the entire group, without significant differences among subgroups (RCT, PBT, Non-R). The cumulative probability for relapse of appendicitis demanding appendectomy was: 0.09, 0.12, 0.12 and 0.13 at 1-, 2-, 3- and 5-year follow-up, with a probability of 0.86 ± 0.013 without appendectomy after 8 years. This may imply an overall benefit of 60–70% by antibiotics during expected 10-year follow-up accounting for initial treatment failures at 10–23% in our published reports.

**Conclusion:**

Antibiotic treatment is safe and effective as a first-line therapy in unselected adults with acute appendicitis with a risk around 15% for long-term relapse following favourable initial treatment response.

## Introduction

Antibiotic treatment of acute appendicitis as alternative to surgical appendectomy is a well-recognized possibility, with favourable response rates of 0.77–0.91 according to randomized and consecutively evaluated patients [1–6]. However, it is still a controversy to what extent antibiotic treatment should be offered systematically as a first-line therapy [7, 8]. It has even been doubted whether antibiotics offers significant resolution of inflamed appendices [9]. This uncertainty is probably in part dependent on that long-term results are essentially lacking in the literature, although satisfying acute and long-term outcomes are reported in both adults [3] and children [10, 11] with uncomplicated appendicitis. The resolution of pain and clinical signs of inflammation was around 75–90% of treated patients depending on clinical stage and selection of patients [1, 2, 12]. The choice of antibiotics is also important, which may vary between countries and across time in different geographic areas [13]. In the present study, we present long-term results on outcome that should encourage physicians to choose antibiotic treatment of acute appendicitis as a safe and evidence-based alternative to acute surgical operations [14].

## Materials and methods

We have earlier published CONSORT flow sheets and inclusions of patients who started on antibiotics (*n* = 561) due to acute appendicitis in either randomized (RCT; *n* = 106) or population-based trials (PBT; *n* = 442) [1, 2]. We also include unpublished subsequently treated non-randomized patients (Non-R; *n* = 271) with favourable initial response to antibiotics. All our patients were offered antibiotic treatment as an alternative to surgery for treatment of assumed acute appendicitis according to clinical evaluations as described and discussed elsewhere [2]. In our published reports, all patients older than 18 years with assumed appendicitis were eligible for inclusion (RCT, PBT). Acute appendicitis was diagnosed according to established practice: the attending physician decided based on disease history, clinical status, computed tomography and gynaecological examination when deemed necessary. Patients were randomized according to birth date (RCT). Evaluation was performed according to intention to treat and per protocol [1]. All patients with suspected appendicitis were invited to have the antibiotics-alone treatment option according to the PBT protocol; with analyses of intention to treat and per protocol as well [2]. All our patients allocated to antibiotics could have surgery without any predetermined specification (required by ethics) if the surgeon in charge deemed it necessary; or if the patient preferred initial operations. Similarly, patients allocated to surgery could choose antibiotics as their first choice. In the RCT study, 369 patients were randomized: 202 were allocated to antibiotics and 167 to surgery; 119 patients started on antibiotics; and 250 had immediate surgery [1]. In the PBT study, a total of 558 consecutive patients were hospitalized and treated due to acute appendicitis: Seventy-nine per cent (442) received antibiotics as their first-line therapy and 20% (*n* = 111) had or preferred primary surgery; 77% on primary antibiotics recovered, while 23% had subsequent appendectomy due to failed initial resolution on antibiotics. The third group (Non-R, *n* = 271) of included patients were informed to have antibiotic treatment by physicians in charge on the same premises when deemed reasonable according to encouraging results in our RCT and PBT studies. These patients have thus been subsequently selected from the same patient population as used for inclusion of patients to the RCT and PBT studies, but without any specific selection criteria beyond a patient offer to try antibiotics as a first-line treatment option. They were clinically judged and evaluated in the same way as patients in RCT and PBT groups [1, 2].

Major patient inclusions were between 2005 and 2013. A majority of presently evaluated randomized patients (*n* = 97) were treated at Sahlgrenska University Hospital, included 2006–2007, while population-based inclusions were more equally performed at Sahlgrenska hospital (*n* = 182) and the Östra University Hospital (*n* = 160) during 2009–2010 according to protocols described in details elsewhere [1, 2]. Non-randomized patients were treated only at Sahlgrenska hospital (*n* = 271) with major inclusions 2010–2013. The Non-R group represented around 20% of all patients treated for acute appendicitis at Sahlgrenska hospital according to a hospital incidence of acute appendicitis around 0.055% per year. Accordingly, we present the results as related to the protocols of our earlier studies and patient inclusions with different study design and inclusion premises; altogether 710 out of 832 patients who were offered and started on antibiotics. The difference of 122 patients was acute treatment failures (15%) by antibiotics. The criteria of treatment failure for antibiotic treatment alone were the same for all groups (RCT, PBT, Non-R); i.e., the need of surgery according to the physicians in charge based on overall clinical evaluation during observation of the patients. Patients who did not recover acutely on antibiotics during their hospital stay (1–2 days) or relapsed within 14 days from onset of appendicitis despite antibiotic treatment were regarded acute treatment failures. Thus, patients who responded favourably to initial antibiotic treatment and left the hospitals with complimentary oral antibiotic treatment for additional 8–10 days were eligible for the present follow-up analysis by a combination of personal letter request to all patients and computer-based search to what extent patients had been operated for appendicitis in any of our hospitals, confirmed by patient file examinations until December 2015. Independently, two research nurses filled in database information on all required aspects.

Randomized patients were treated with cefotaxime 1 g twice and metronidazole 1.5 g once for at least 24 h and in some cases within 36 h [1]. Oral continuation was ciprofloxacin 0.5 g twice daily and metronidazole 0.4 g three times per day. The population-based study used piperacillin plus tazobactam 4 g every 8 h for at least three occasions usually within 24–36 h, while oral continuation was ciprofloxacin and metronidazole [2]. This treatment was also used for non-randomized patients, all in agreement with licensed physicians for infection disease considering local resistance pattern to antibiotics [13].

Table 1 shows clinical characteristics of all patients in the present evaluation at start and before their initial treatment with antibiotics in previous trials and non-randomized treatments.

**Table 1:** Clinical characteristics of 710 patients at their start of antibiotic treatment for acute appendicitis in prospective randomized (RCT) population-based trial (PBT) and non-randomized treatment (Non-R)

	RCT (97)	PBT (342)	Non-R (271)	*p*<
Age (years)	39.6 ± 1.8	33.5 ± 1.2	37.4 ± 1.0	0.001
CRP (mg/L)	51 ± 6	44 ± 3	51 ± 3	Ns
WCC (10^−9^/L)	12.0 ± 0.4	12.9 ± 0.3	13.4 ± 0.6	Ns
Body temperature (C)	37.1 ± 0.5	37.1 ± 0.6	36.9 ± 0.7	0.01

Body temperature is mean ± SD; Age, CRP, WCC are mean ± SEM; Number of patients within parenthesis

There was no difference between male (*n* = 340) and female (*n* = 370) patients among the groups

*CRP* is C-reactive protein

*WCC* is leucocyte cell count

### Statistics

Results are presented as standard statistics (mean, median, SEM, SD) as indicated in tables. Multigroup comparisons were performed by ANOVA. Median values were compared by Kruskal–Wallis nonparametric test. Relapse of appendicitis is regarded a positive statistical event (uncensored), while uneventful healthy post-treatment periods are regarded censored events in Log rank analysis to obtain time course probabilities for relapse of appendicitis demanding surgical appendectomy. Prediction of relapse and time to relapse were evaluated by multivariate and logistic regressions.

## Results

According to our present selection criteria, 710 patients were available in our database for long-term follow-up from our previous randomized and population-based trials as well as non-randomized treatments. Clinical characteristics such as age, blood C-reactive protein (CRP), leucocyte counts (WCC) and body temperature at the start of antibiotic treatment were in large comparable among randomized and non-randomized patients, which was also true for females versus males in subgroups of patients (Table 1). There were small significant differences in age and body temperature among the groups at the start of antibiotic treatment (Table 1). The number of males (*n* = 340) versus females (*n* = 370) on antibiotic treatment did not differ significantly (*p* < 0.81); being 35 ± 1 and 36 ± 1 years old, respectively.

The overall follow-up range was 3495 days (9.57 years) for all patients; between 2 and 3497 days. The overall range until relapse and appendectomy for all patient groups was 1972 days (range 2–1974) and 221 days in median with insignificant differences in time to relapse among the subgroups of patients (Table 2). Clinical characteristics (age, CRP, WCC, body temperature: Table 1) did not predict time to relapse and appendectomy.

**Table 2:** Time to relapse of appendicitis and total observation time on group basis in patients on primary antibiotic treatment and surgical appendectomy as secondary treatment

	RCT	PBT	Non-R	*p*<
Days until op				
Mean	296 ± 57	378 ± 90	315 ± 62	0.86^a^
Median	220	274	152	0.20^b^
Range	681	1903	1878	
Total observation time without op (days)				
Mean	3252 ± 15	2264 ± 6	1551 ± 26	
Median	3232	2266	1570	
Range	488	284	1424	

Range is the difference between maximum and minimum time in days, between 2 and 3497 days for all 710 patients. Observation time is not compared statistically since patients were recruited during different periods, 2005–2006; 2009–2010; 2010–2013

^a^Tested by ANOVA

^b^Tested by Kruskal–Wallis nonparametric test

**Fig. 1:**
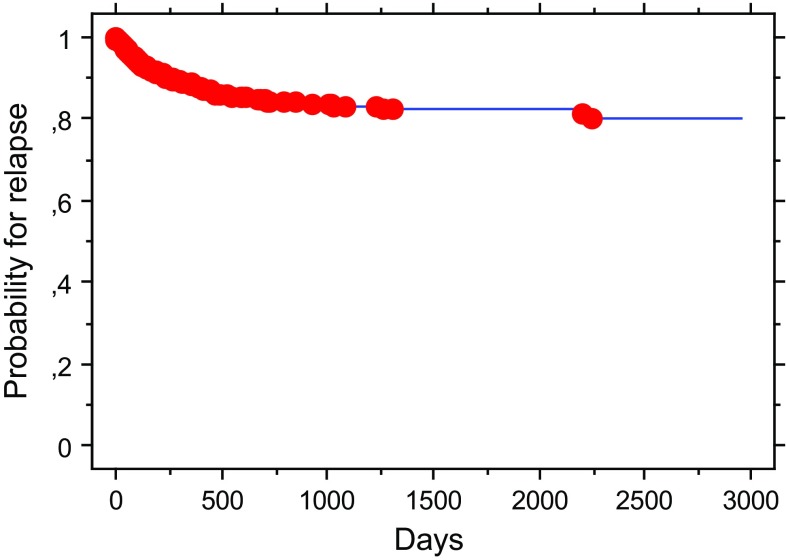
Time course probabilities for relapse of appendicitis confirmed at appendectomy in 710 patients who experienced an initial favourable treatment response to antibiotics for their acute appendicitis in randomized (RCT, *n* = 97) and population-based studies (PBS, *n* = 342) as well as in non-randomized (Non-R, *n* = 271) treatments as described in Materials and methods. The slopes for the three patient groups (RCT, PBS, Non-R) were similar (not shown)

Figure 1 shows the time course probability for relapse of appendicitis demanding appendectomy, with insignificant variation among patients in different clinical trials and non-randomized treatments (not shown). Most recurrent appendicitis occurred within 1 year. The cumulative probability for relapse demanding appendectomy was: 0.09, 0.12, 0.12 and 0.13 at 1, 2, 3 and 5 years, respectively. This corresponds to an observed long-term cumulative probability of 0.86 ± 0.013 to avoid operation across 8 years following an initial favourable response to antibiotic treatment. The number of operated patients during follow-up was: 16 (RCT), 29 (PBT) and 48 (Non-R). Thus, available information in our database predicts that the expected overall benefit of antibiotic treatment of acute appendicitis should be around 60–70% within 10-year follow-up including initial failures between 10 and 23% in unselected adults without serious complications due to antibiotic treatment. Serious complication (bowel obstruction, wound rupture, hernia, pulmonary embolism, cardiac problems, ileocecal resection, caval thrombosis) did only occur following appendectomy according to our records as reported [1, 2] Our Non-R patients showed only minor complications related to antibiotics such as gastrointestinal discomfort. Our previous publications have indicated similar complications among patients subjected to primary or secondary appendectomy [1, 2].

## Discussion

We have earlier reported that antibiotic treatment of acute appendicitis is safe and was associated with significantly less complications compared to acute surgical interventions [1, 2], although conventional open appendectomy was not compared specifically to laparoscopic extirpation in our earlier trials [15]. Previously published studies on antibiotic treatment for acute appendicitis have mostly included only patients with confirmed positive imaging, which is a kind of restricted patient selection. By contrast, our previous studies included more or less unselected adult patients (>18 years old) with “assumed appendicitis” (both complicated and uncomplicated) according to Swedish standard criteria based on anamnesis, clinical and abdominal examinations, clinical chemistry as well as imaging in patients where the clinician in charge deemed it necessary for a high probability of positive diagnosis (in 63% of all our patients). Correct diagnoses of acute appendicitis in our hospital in unselected patients are close to 90% specificity, which is a level that applications with CT scans or ultrasound in all patients do not improve under most circumstances, particularly not in acute or emergency patients. Thus, our approach to include unselected patients based on overall clinical judgments should not be regarded a weakness of our protocols. We remain strong in the view that our results reflect standard clinical conditions at least in Scandinavian countries. This conclusion is particularly supported by the fact that RCT, PBT and Non-R studies indicate similar results at follow-up, which is as good as clinical studies can provide.

The role of imaging (CT, MR, ultrasound) for positive diagnosis may well be a matter of continuous debate among clinicians, although overall diagnostics usually leave a window of uncertain opportunities between 85 and 100% specificity [16–18]. We want to emphasize this fact, to avoid concerned discussions about optimized diagnosis of acute appendicitis, which is still an unsettled matter, although trained physicians usually manage to predict a correct positive diagnosis between 90 and 95% [1, 19, 20]. It is also important to remind that most large hospitals may provide treatment results with negative findings of appendicitis at laparotomy for assumed acute appendicitis (false positive diagnosis) around 5–10% as reported [1–3]. Thus, we have never tried to select patients, based on clinical or imaging evaluations, with signs of perforated (complicated) or non-perforated (uncomplicated) appendix before offering patients antibiotic treatments. Thus, all our patients were close to unselected, with confirmed or suspected perforations around 20–30% at subsequent appendectomies [1, 2].

Our previous results on antibiotic treatment of acute appendicitis have indicated that initial and short-term results are good or even very good [12], depending on personal preferences. This means that around 85% of all patients could leave our hospitals without operation, which saved a considerable amount of money without particular harm and risks compared to surgical appendectomies [15]. This perspective and fact have been difficult to accept and cope with for groups of physicians, particularly surgeons, who tend to conclude that appendectomy is a “superior” method since relapse will never occur [7, 8]. However, such arguments are hampered by the fact that serious complications following surgical appendectomy remain significant matters and may sometimes create long-life problems such as hernia, conditions with intestinal obstruction demanding reoperations and sometimes even intestinal strangulation [3, 21]. These facts should be considered in the light that we did not observe any serious complications due to antibiotic treatment besides diarrhoea and minor allergic skin reactions during treatment with antibiotics [1, 2]. Eventual risk differences for effects on fertility in females on antibiotic treatment versus surgical appendectomy are presently unknown but may be a question in future investigations. Besides, it is our experience that a large number of patients are highly willing to try antibiotic treatment, both once and several times, before definite operations when provided current and available evidence-based information in published reports, as also observed by others [14]. Anyway, an important finding in our previous studies is that immediate start of antibiotic treatment of patients with suspected acute appendicitis is without negative matters, besides the fact that operation may soon be necessary in about 10–20% of the patients depending on patient selection criteria [12].

With all above perspectives and eventual hesitance despite previous promising reports, it appears now that antibiotic treatment of patients with acute appendicitis is a long-term safe and effective treatment [3, 4]. This means that around 85% of all patients who left the hospital with initially overwhelming relief of symptoms and clinical recovery did not experienced appendectomy within a median follow-up time close to 6 years. According to the slope for uncensored events (appendectomy), it seems unlikely that recurrences will reappear at significant rates beyond 8-year observation. Therefore, one may estimate that antibiotic treatment of “assumed acute appendicitis” may be definite in 60–70% of unselected adult patients with acute appendicitis including initial treatment failures (10–23%). It can then be predicted that around 55% of all unselected patients with “true” appendicitis should be cured for at least 8–10 years following onset of appendicitis, considering that a maximum of 7–10% of patients with “assumed appendicitis” may have other benign explanations behind their acute abdominal symptoms [1]. A question may, however, remain to what extent a significant number of our patients, that were successfully treated by antibiotics, may have shown such favourable results without any treatment intervention at all [22–24]. Our previous and present results may now legitimate such investigations from ethical perspectives, with antibiotic and surgical interventions as alternatives to observational expectation, where patients could be selected for treatment alternatives according to various algorithms [12, 24].
